# Alleviation of cadmium stress in rice by inoculation of *Bacillus cereus*

**DOI:** 10.7717/peerj.13131

**Published:** 2022-05-02

**Authors:** Zahra Jabeen, Faiza Irshad, Ayesha Habib, Nazim Hussain, Muhammad Sajjad, Saqib Mumtaz, Sidra Rehman, Waseem Haider, Muhammad Nadeem Hassan

**Affiliations:** 1 Department of Biosciences, COMSATS University Islamabad, Islamabad, Pakistan; 2 Zhejiang University, Institute of Crop Science, Zhejiang Key Laboratory of Crop Germplasm, Hangzhou, PR China

**Keywords:** Cadmium, Rice, *Bacillus cereus*, PGPR, ROS

## Abstract

Heavy metal resistant bacteria are of great importance because they play a crucial role in bioremediation. In the present study, 11 bacterial strains isolated from industrial waste were screened under different concentrations of cadmium (Cd) (100 µM and 200 µM). Among 11 strains, the Cd tolerant *Bacillus cereus* (S_6_D_1–105_) strain was selected for *in vitro* and *in vivo* studies. *B. cereus* was able to solubilize potassium, and phosphate as well as produce protease and siderophores during plate essays. Moreover, we observed the response of hydroponically grown rice plants, inoculated with *B. cereus* which was able to promote plant growth, by increasing plant biomass, chlorophyll contents, relative water content, different antioxidant enzymatic activity such as catalase, superoxide dismutase, ascorbate peroxidase, polyphenol oxidase and phenylalanine ammonia-lyase and reducing malondialdehyde content in both roots and leaves of rice plants under Cd stress. Our results showed that the *B. cereus* can be used as a biofertilizer which might be beneficial for rice cultivation in Cd contaminated soils.

## Introduction

In the present era, heavy metal stress is growing at a frightening level, and it has become a universal concern. Abiotic stresses such as drought, salinity, and metals are among the most deteriorating factors that hinder crop growth and yield (Naveed et al., 2014). Although the soil is a natural origin of heavy metals, several anthropogenic and geologic activities intensify their concentration to lethal levels for plants as well as animals (Chibuike & Obiora, 2014). Globally, mineral problems are affecting almost 60% of the agricultural lands. Among them, Cd is regarded as a well-known deleterious environmental pollutant payable to its pronounced toxicity, high water solubility, and high mobility that readily takes it up from the soil to plant roots (Rajamoorthy & Munusamy, 2015). In most soils, the natural presence of Cd is less than 1 mg/kg but its value is increased beyond permissible limits due to incessant application of wastewater from industries. Human activities result in an annual release of approximately 13,000 tons of Cd into the surroundings (Kumar et al., 2019). In plants, Cd triggers the synthesis of reactive oxygen species, hinders uptake, utilization and transport of water and essential nutrients as well as modifies photosynthetic machinery, hence resulting in plant death (Qadir et al., 2016). Usually, Cd accumulates in crop’s edible parts and its toxic nature can cause lipid membrane instability and chlorosis owing to plant growth and is eventually delivered to humans by food chain (Chen et al., 2016). The bioaccumulation of Cd in the food chain and the human body results in acute and chronic health effects (Bakshi et al., 2018).

Rice is Pakistan’s second main staple food and is also the primary food source of nearly half of the global population. Rice plant also acts as a Cd accumulation model for monocot species (Chiu et al., 2015). The renal tubular syndrome has been reported in many people after rice consumption cultivated on Cd-polluted lands (Ahmad et al., 2014). Rice has the capability of taking up Cd from soils due to induction of oxidative stress (He et al., 2017), making Cd a most concerning abiotic stress limiting rice productivity. Even though plants are known to possess various mechanisms to defend the heavy metals intoxications but the failure of these strategies beyond definite limits endangers the plant’s survival (Clemens & Ma, 2016). Hereafter, removal of accumulated metals becomes indispensable for normal plant functioning as well as for the protection of plant dependent species. The practices being utilized for heavy metals cleanup from polluted soils comprise *in situ* fixation (change of metals state to a form which plants cannot absorb by addition of chemicals), excavation (physical elimination from polluted land), and soil washing. Still, these procedures are costly and less proficient (Mishra, Singh & Arora, 2017). Increased or decreased uptake of trace elements in plants resulting from more or less bacterial heavy metals bioavailability has been revealed in several studies (Chen, Muckerman & Fujita, 2013). Application of heavy metal resistant plant growth promoting bacteria (PGPB) under harsh circumstances may maintain plant growth as they solubilize phosphate, fix atmospheric nitrogen, and produce growth promoting phytohormones or siderophores. These characters are predominantly vital for the supportive growth of plants under stress (Etesami & Maheshwari, 2018). Soil microbes may alter metal availability in the soil solution by releasing certain chelating elements to acidify the soil (Colombo, Raposo & Théry, 2014). The metabolic competencies of microorganisms sustained by their molecular machinery enable them to perform even under higher concentration of heavy metals (Basu & Bundick, 2017).

The objective of this study was to screen bacteria isolated from industrial waste with Cd tolerance ability and utilization of these strains in fighting Cd stress in rice plants to improve its growth under Cd stress and hence to fulfill safe food demands of a globally increasing population.

## Materials and Methods

### Revival of industrial waste bacterial strains

Eleven bacterial strains isolated from industrial waste were obtained from the Applied Microbiology and Biotechnology Lab, COMSATS University, Islamabad, Pakistan. Revival of these bacterial strains from their glycerol stocks was made on Luria Bertani (LB) media: NaCl (5 mg), Tryptone (5 g), Agar (7.5 g), and Yeast (2.5 g) in 500 ml distilled water. Streaking of bacterial strains was performed on each plate containing 20 ml agar media. After streaking, the plates were secured with parafilm to avoid any contamination. Petri plates were then placed in an incubator at 30 °C for approximately 24 h. Afterward separate bacterial colonies were obtained on plates and stocked at 4 °C for further use.

### Screening of industrial waste bacterial strains for cadmium tolerance

Cadmium (in the form of CdCl_2_) tolerance of bacterial strains was assessed by growing bacteria in nutrient agar media with different concentrations of CdCl_2_ (100 µM and 200 µM). Control plates were also maintained. Eleven different strains *i.e*. S_3_D_1–1_, S_3_D_1–4_, S_3_D_4–6_, S_1_D_2–33_, S_1_D_2–34_, S_6_D_1–42_, S_1–89_, S_6_D_1–105_, S_2_D_2–111_, R_1_, and R_2_ were used. Streaking of these strains was performed afterwards on agar plates and incubated for 48 h at 30 °C. Growth of the tested strains was observed under both the concentrations of CdCl_2_ to screen tolerant bacterial strains.

### Determination of minimum inhibitory concentration (MIC) of bacteria for Cadmium

Maximum resistance of the selected bacterial isolate S_6_D_1–105_ against the increasing concentrations of CdCl_2_ on nutrient agar plates was evaluated until the strain was unable to grow colonies on the LB agar plates. Nutrient Agar plates augmented with CdCl_2_ stress at 100 µM to 2,000 µM concentrations were used to determine MIC.

### DNA extraction

For genomic DNA extraction, Cetyltrimethylammonium Bromide (CTAB) based procedure was used. Samples were merged with 500 μl of CTAB buffer, 300 μl sterile dH_2_O, and 20 μl proteinase K (20 mg/ml) along with 1% polyvinylpyrrolidone (PVP). Afterwards, samples were incubated at 65 °C for 2 min. The blend was then incubated in a water bath at 65 °C for 10 min. The samples were then centrifuged at 16,000×*g* for 10 min along with the extraction of supernatant twice with 500 μl chloroform. The upper layer was collected and incubated for 1 h at room temperature, thereafter, merging it with the twofold volume of CTAB solution. The samples were further centrifuged at 16,000×*g* for 5 min. The pellet was mixed in the mixture of 350 μl chloroform and 1.2 M NaCl, and centrifuged for 10 min at 16,000×*g*. Again, the upper layer was shifted to a new test tube, blended with isopropanol, and further centrifuged for 10 min at 16,000×*g*. Subsequently, supernatant was discarded, and pellet was washed with 500 μl of 70% ethanol. Following centrifugation, the supernatant was thoroughly disposed, and the pellet was dried up and was dissolved in 100 μl ddH_2_O.

### Identification of bacteria by 16S rRNA gene sequencing

The 16S rRNA gene of bacteria was amplified using universal primers as, F-primer MP1 (5’-CGGGATCCAGAGTTTGATCCTGGTCAGAACGAACGCT-3’) and R-primer MP6 (5’-CGGGATCCTACGGCTACCTTGTTACGACTTCACCCC-3’). Amplification was carried out by PCR with a reaction mixture of 25 µl, that consisted of PCR water, MgCl_2_, 10× Taq buffer, Taq polymerase 5 U/µl, reverse primer 10 µM, forward primer 10 µM, dNTPs 10 mM, and DNA template ≥1,000 ng. The PCR was carried out in a thermal cycler manipulating cycling condition, that contained 1 cycle of initial denaturation at 95 °C for 5 min, then 35 cycles of denaturation for 45 s at 95 °C, annealing at 54–65 °C for 45 s and extension for 1 min 30 s at 72 °C for 16S rRNA gene and final extension was executed for 10 min at 72 °C. The PCR products were assessed by 1% agarose gel electrophoresis. The bacterial 16S rRNA gene sequence was identified using the Basic Local Alignment Search Tool (BLAST) algorithm at the National Centre for Biotechnology Information (NCBI) and accession number (OK310861) of the most potent bacterial sequence submitted to NCBI Gene Bank was obtained, accordingly.

### Plant growth promoting traits

Plant growth promoting (PGP) traits such as: phosphate solubilization, potassium solubilization, as well as protease and siderophores production by Cd tolerant bacterial strain were examined.

### Phosphate solubilization assay

The bacterial strain was tested for phosphate solubilization by an agar assay method using Pikovaskaya’s media. The bacterial strain was streaked on Pikovaskaya’s plates, and then placed in an incubator for 7 days at 30 °C. After incubation period, plates were observed for the presence of clear halo-zones surrounding the culture spot which points towards the isolate’s phosphate solubilization ability. The solubilization index was accessed by the following formula:

$\rm{SI} = {\rm{colony \ diameter} + \rm{halozone \ diameter} \over \rm{ Colony \ diameter}}$

### Potassium solubilization assay

The bacterial strain was tested for potassium solubilization using Aleksandrov media. The media was dispensed into petri plates. After the media was solidified, the bacterial strain was streaked on the media. Plates were incubated for 3 days at 30 °C. After incubation, the presence of clear halo-zones surrounding the culture spot, indicated the potassium solubilization potential of the bacteria. Potassium solubilization index was noted by the following formula:

$\rm{SI} = {\rm{colony \ diameter} + \rm{halozone \ diameter} \over \rm{ Colony \ diameter}}$

### Protease detection

The protease production test was performed according to the method mentioned by Hirano & Nagao (1988). Petri plates with nutrient agar medium altered with skimmed milk powder (1%) were used for protease detection. The 24 h bacterial colonies were spot inoculated on media plates and kept for 48 h in an incubator. Bacterial isolate having a clearing zone formation around the colony was taken positively for protease enzyme production (Kumar et al., 2012).

$\rm{SI} = {\rm{colony \ diameter} + \rm{halozone \ diameter} \over \rm{ Colony \ diameter}}$

### Production of siderophores

Bacterial isolate was observed for siderophores production by nutrient agar medium added with an indicator dye. The nutrient agar media containing the bacterial culture was incubated for 7 days at 30 °C. The positive or negative reaction of bacterial strain to the assay was recorded. After incubation, plates were detected for halo-zones formation and the following formula for accessing the solubilization index was used:

$\rm{SI} = {\rm{colony \ diameter} + \rm{halozone \ diameter} \over \rm{ Colony \ diameter}}$

### Screening of rice genotypes for cadmium tolerance

Seeds of three rice genotypes were acquired from Rice Research Institute (Kala Shah Kahu), District Shaikhupura Punjab, Pakistan. Fifty seeds of each genotype were soaked for 3 min in H_2_O_2_ (3%) and were washed with distilled water. These seeds were grown in the germination box on wet double-layer filter paper. Seeds were germinated under different levels of Cd concentration (100 µM, 200 µM, and 300 µM). Seeds were kept in germination boxes in the dark until the plumule (shoots) and radical (roots) of the seeds emerged. After 7 days of germination fresh and dry weights, shoot, and root lengths were measured.

### Pot experiment

After screening, test seeds of Super Basmati rice with high germination percentage were selected for further experimental work. After 7 days of germination, seeds of Super Basmati were grown hydroponically in Hoagland nutrient solution in plastic pots containing foam-plugged holes placed in a greenhouse. Nutrient solution composition at its full strength was mg/L (NH_4_)_2_SO_4_ 48.2, K_2_SO_4_ 15.9, MgSO_4_.7H_2_O 154.88, KNO_3_ 37, KH_2_PO_4_ 49.6, Ca (NO_3_).2H_2_O 172.36, MnCl_2_.4H_2_O 1.8, ZnSO_4_.7H_2_O 0.22, Fe-Citrate 14, CuSO_4_.5H_2_O 0.08, H_3_BO_3_ 5.8, H_2_MnO_4_ 0.02 and prepared according to Jabeen et al. (2021). The pH of the nutrient solution was maintained at 6.0 ± 0.5 with HCl or NaOH. Bacterial strain S_6_D_1–105_ was pre inoculated with 2 weeks old rice seedlings. After 5 days of inoculation, rice plants were exposed to four treatments *i.e*., (1) Control, (2) 100 µM CdCl_2_ stress, (3) bacterial strain-105, (4) 100 µM CdCl_2_ + bacterial strain-105 with three replicates.

After 14 days of treatment, rice plants were harvested. Plant roots, and leaves were separated for assessment of different growth parameters *i.e*. roots and shoots length, fresh and dry weight, leaf relative water content, chlorophyll content, and antioxidant enzymatic activities. Roots and leaves were collected and frozen immediately in liquid nitrogen and stored at –80 °C until further processing.

### Plant morphological traits

Shoots and roots length of rice was measured. The fresh weight of roots and shoots from each treatment was recorded. For dry weight analysis, plant samples were dried for 72 h at 70 °C.

### Root scanning

Roots of all the treated as well as untreated (control) plants were gathered separately and rinsed three times with deionized water. Roots taken from each treatment sample were spread carefully in a transparent tray containing distilled water and scanned with Epson Expression 16801.0 scanner for analysis.

### Chlorophyll contents

Fresh leaves of rice (0.2 g) were homogenized with mortar and pestle in 20 ml (80%) acetone and samples were placed in dark for 24 h. After that, samples were centrifuged for about 10 min and Chlorophyll contents were measured by taking supernatant. Chlorophyll-a, chlorophyll-b, and carotenoids were checked by recording the corresponding absorbance at 470 nm, 647 nm, and 664 nm respectively. The quantity of photosynthetic pigments was expressed in mg/g of the fresh weight (FW) (Biczak, 2016).

### Determination of malondialdehyde (MDA)

Fresh rice leaves (0.5 g) were homogenized in 5 ml (0.1%) trichloroacetic acid (TCA). Malondialdehyde (MDA) determination was carried by measuring the absorbance of supernatant at 532 nm and 600 nm and it was calculated according to its extinction coefficient of 155 nm/cm (Biczak, 2016).

### Superoxide dismutase assay (SOD)

Superoxide dismutase (SOD) activity was measured by its ability to inhibit photochemical reduction of NBT on blue formazan, and then monitored by the absorbance of assay mixture spectrophotometrically at 560 nm (Habib, Kausar & Saud, 2016).

### Catalase (CAT) activity

Activity of catalase (CAT) in the leaves and roots of rice was assessed with spectrophotometer at 30 °C by monitoring the absorbance resulting from H_2_O_2_ decomposition at 240 nm_._ The CAT activity was measured in accordance with protocol followed by Habib, Kausar & Saud (2016).

### Peroxidase (POD) activity

Peroxidase (POD) activity was determined by analysis of POD ability for oxidizing guaiacol using H_2_O_2_. Reaction mixture was composed of 2.5 ml of 50 mmol/L potassium phosphate buffer (pH 6.1), 1 ml (1%) hydrogen peroxide, 1 ml (1%) guaiacol and 10–20 μl enzyme extract (Marszałek, Mitek & Skąpska, 2015).

### Ascorbate peroxidase (APX) assay

Ascorbate peroxidase (APX) activity was measured according to Habib, Kausar & Saud (2016). Three ml reagent solution containing potassium-phosphate (50 mM) buffer (pH 7.0), H_2_O_2_ (0.1 mM), ascorbate (0.5 mM), and enzyme extract (20 μl) was used. The APX activity was observed at 290 nm, using the extinction coefficient (2.8 mM/cm).

### Polyphenol oxidase (PPO) activity

Determination of polyphenol oxidase (PPO) activity was carried out by adding 100 μL of the enzymatic extract into 3 mL catechol (0.07 M) in sodium phosphate buffer (0.05 M) having pH 6.5. Assay mixture absorbance was measured in the kinetic mode at 420 nm (25 °C for 10 min) using the UV-visible spectrophotometer (Zareei, Javadi & Aryal, 2018).

### Phenylalanine ammonia lyase (PAL)

PAL activity was determined according to Gill et al. (2016). The reaction mixture contained 3 mM/L-phenylalanine, 150 mM of tris-HCL and enzyme extract. PAL activity was measured at 290 nm. PAL activity was measured by using formula:

${\rm{PAL \ Activity }}\ ({\rm{U/ml)}} = {{\rm{OD270}} - {\rm{OD270 \ Blank}} \times  {\rm{a}} \times {\rm{df}} \over {19.73 \times 0.1}}$

where, 3 represents total volume of assay, 19.73 represents milli-molar extinction co-efficient, and df represents dilution factor.

### Relative water content (RWC)

Relative water content of rice leaves was determined according to Weatherley (1950). Fresh weight of control and treated plants was measured immediately after harvesting. The leaves were then soaked in distilled water container for 24 h. Then, weight of completely turgid leaves was measured again. The leaves were subsequently dried in a conventional oven to a constant weight at about 60 °C for 72 h. RWC of leaves was measured by the following formula:

${\rm{RWC}}\% = [({\rm{FW-DW}}) / ({\rm{TW-DW}})] \times 100$

FW, fresh weight; DW, dry weight; TW, turgid weight.

### Statistical analysis

Statistical analysis of data was done by applying One-way analysis of variance (ANOVA). Variability in data was expressed as standard error and the assessment of level of significance among different treatments was carried out by Least Significance Difference (LSD) at a significance level of *P* value ≤ 0.05 (Yang et al., 2016). All data are the means ± SD calculated from three replicates per treatment.

## Results

### Screening, identification, and morphology of bacterial strains isolated from industrial waste for CdCl_2_ tolerance

**Figure 1:**
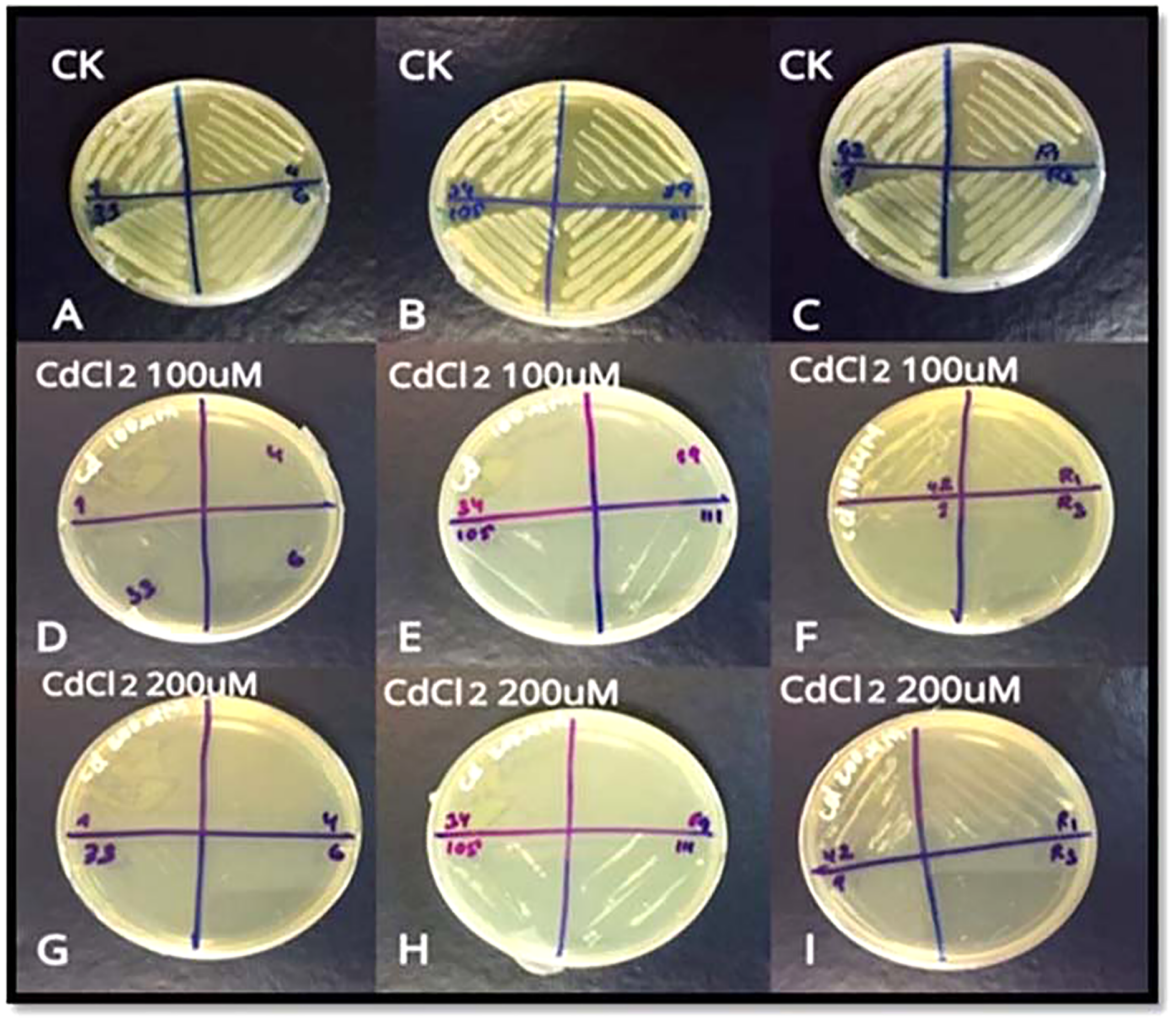
(A–I) Growth of three resistant bacterial strains (S_6_D_1–42_, S_6_D_1–105_ and S_2_D_2–111_) under Cd (100 and 200 µM) stress.

For screening, 11 strains of bacteria *i.e*. S_3_D_1–1_, S_3_D_1–4_, S_3_D_4–6_, S_1_D_2–33_, S_1_D_2–34_, S_6_D_1–42_, S_1–89_, S_6_D_1–105_, S_2_D_2–111_, R1, and R2 were used. Only three strains S_6_D_1–42_, S_6_D_1–105_ and S_2_D_2–111_ showed growth at 100 µM and 200 µM Cd concentrations (Fig. 1A). We selected Cd tolerant S_6_D_1–105_ strain for further experiments and determined its morphological features such as surface, colony shape, elevation edge/margin, size, and opacity (Table 1). Based on 16S rRNA gene sequence analysis, the bacterial isolate S_6_D_1–105_ (OK310861) showed maximum similarity with *B. cereus* (97.30%).

**Table 1:** Morphological characteristics of selected bacterial strain.

Surface	Shape	Margin	Elevation	Opacity	Size (mm)	Texture
Rough and glistening	Irregular	Undulate	Raised	Opaque	<1 mm	Buttery

### Identification of PGPR

#### Minimum inhibitory concentration (MIC) of Cd resistant bacteria

**Figure 2:**
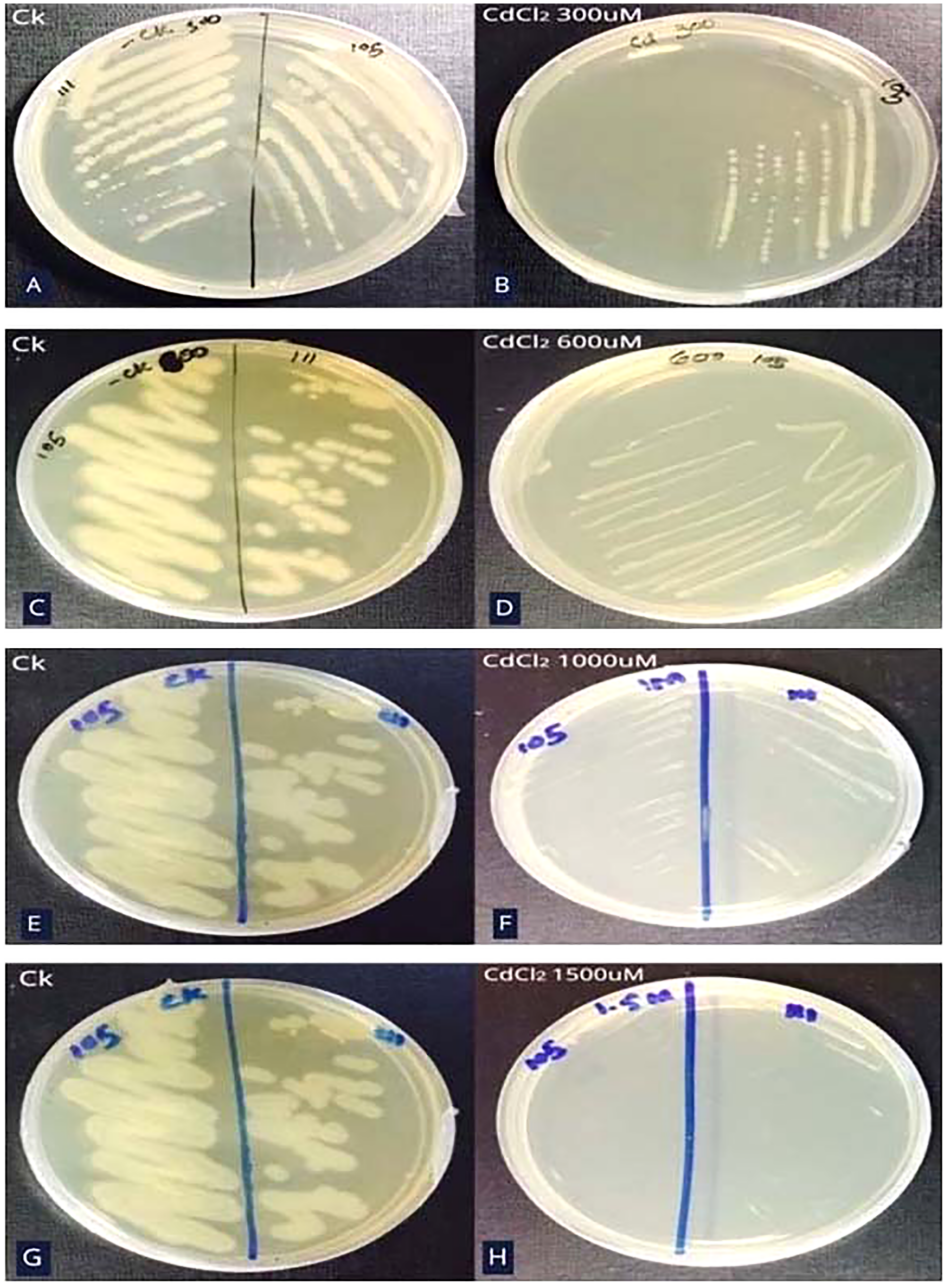
Minimum inhibitory concentration (MIC) of Cd resistant bacteria.

Bacterial strain S_6_D_1–105_ was able to grow up to 1,000 µM CdCl_2_ concentration which was determined by steadily increasing CdCl_2_ concentration on growth media till the colony failed to propagate on agar plates (Figs. 2A–2H).

#### Plant growth promoting (PGP) traits of Cd resistant bacteria

**Figure 3:**
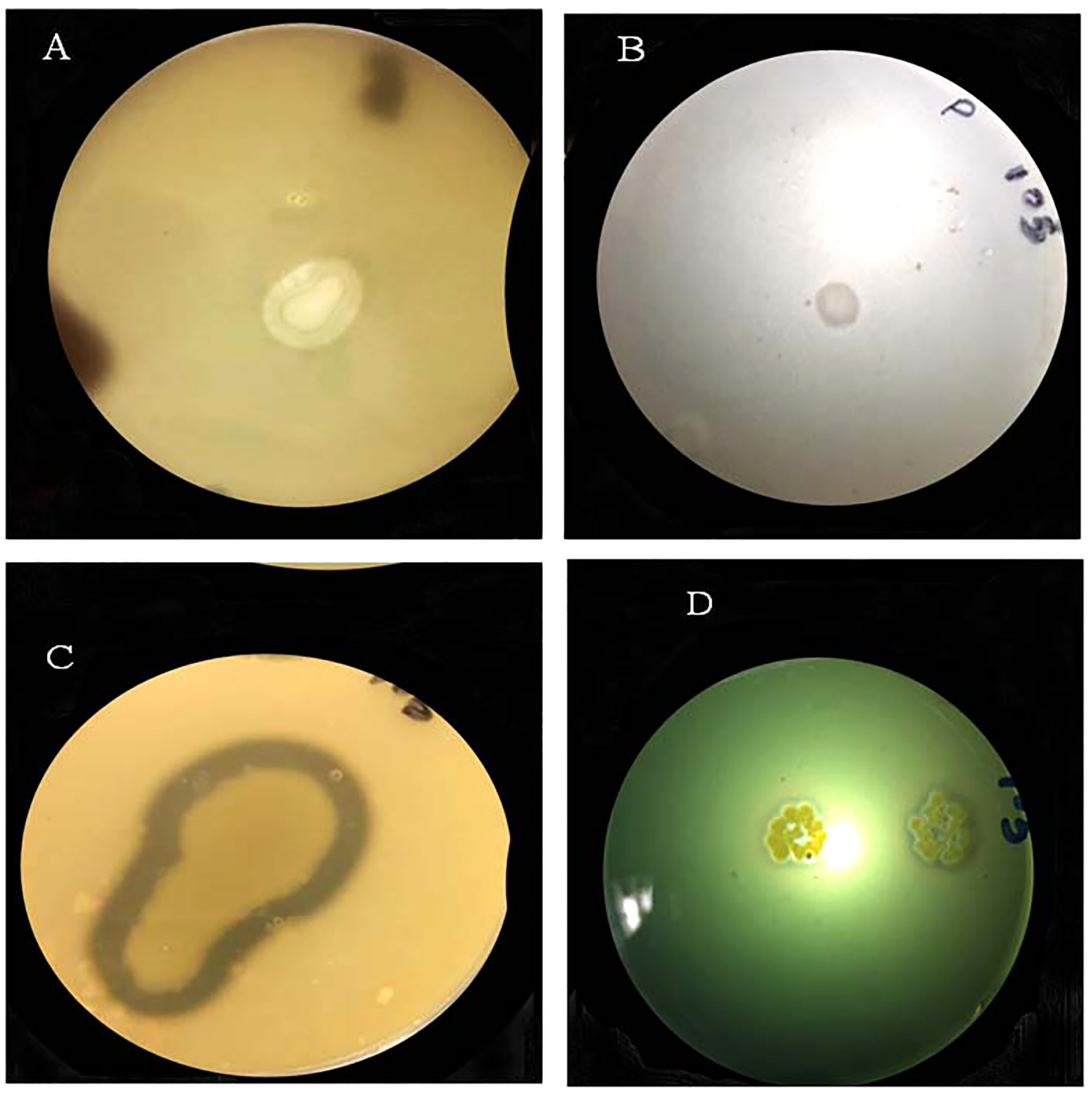
Halo-zone formation during the following plant growth promoting tests: (A) potassium solubilization, (B) phosphate solubilization, (C) protease production, (D) siderophore production.

The bacterial strain S_6_D_1–105_ was capable of protease and siderophores production and phosphate and potassium solubilization in plate-based test, supported by the appearance of a clear halo zone around the bacterial colony. Strains showed positive results for all tested PGP traits (Fig. 3; Table 2).

**Table 2:** Protease, phosphate and potassium solubilization assays of metal tolerant strains.

Strain	PGPR traits	+ive/–ive	Solubilization index (SI)
S_6_D_1–105_	Protease	+ive	2.7 cm
S_6_D_1–105_	Phosphate	+ive	1.2 cm
S_6_D_1–105_	Potassium	+ive	1.1 cm
S_6_D_1–105_	Siderophore	+ive	1.4 cm

#### Screening of rice genotypes for CdCl_2_ tolerance

**Figure 4:**
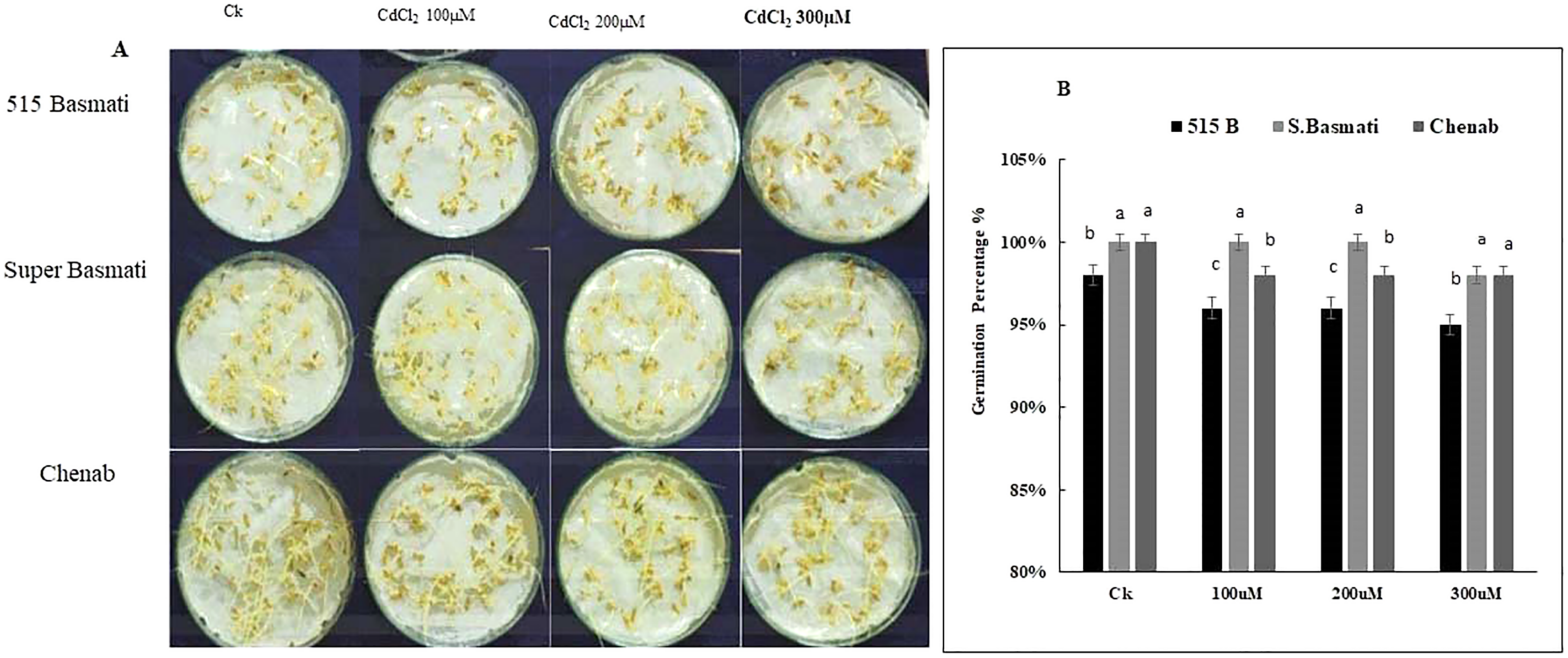
(A) Rice seeds germination percentage, (B) screening of rice genotypes under Cd stress.

Three rice genotypes (Basmati 515, Super Basmati, and Chenab) were exposed to 100 µM, 200 µM, and 300 µM CdCl_2_ stress for 6 days (Fig. 4A). On the sixth day of germination Basmati 515 showed 98% growth under the control condition, 96% growth under 100 µM, 98% growth under 200 µM and 95% growth under 300 µM CdCl_2_ stress, Super Basmati showed 100% growth at control and all three levels of CdCl_2_ stress. Chenab showed 100% growth under control, 98% growth under 100 µM, 200 µM and 300 µM of CdCl_2_ stress (Figs. 4A and 4B). Therefore, we selected Super Basmati for further pot experiments.

#### Growth and biomass of rice inoculated with bacterial strain S_6_D_1–105_ under cadmium stress

**Figure 5:**
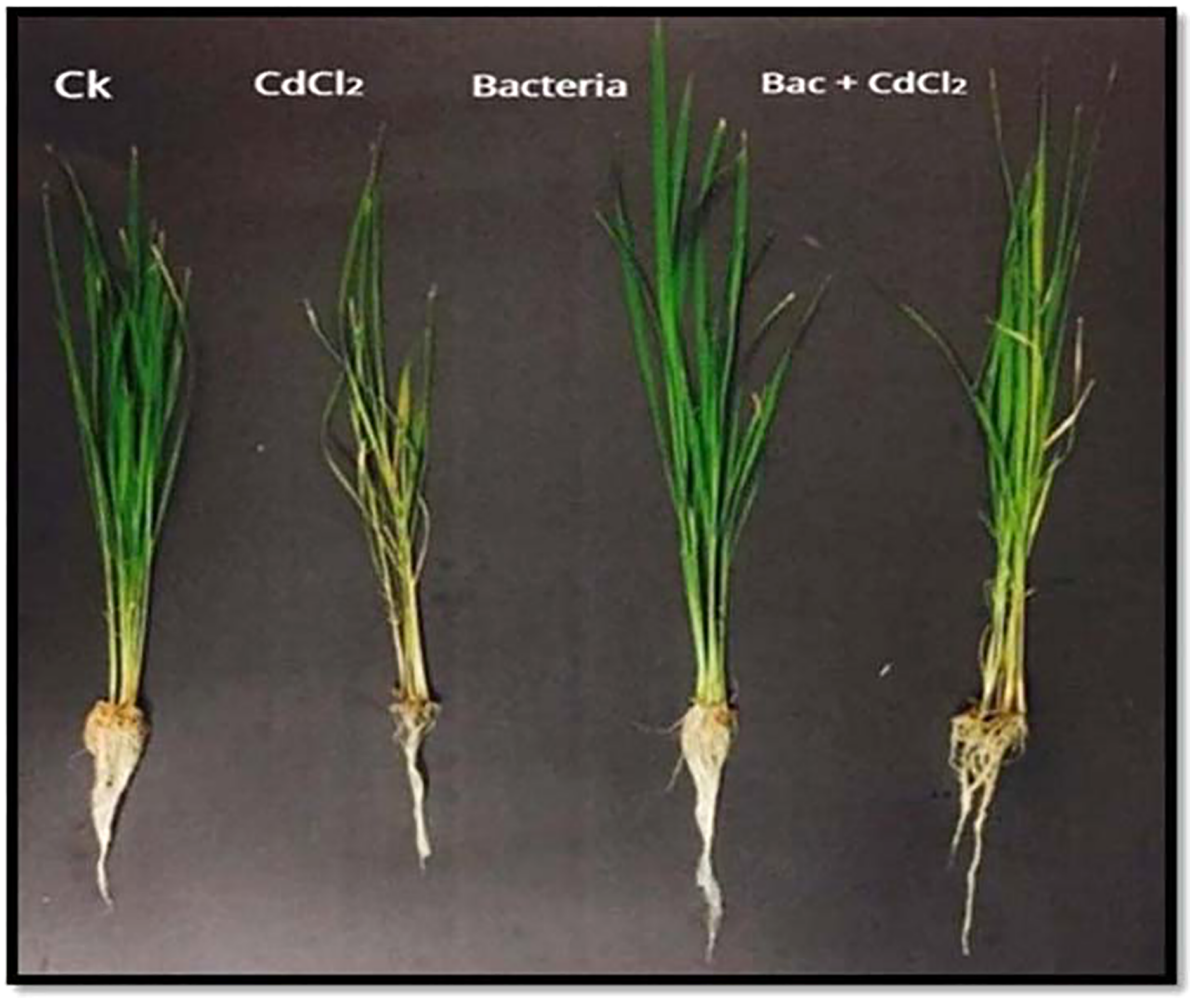
Effect of *B. cereus* S_6_D_1–105_ and Cd stress on the shoots and roots length of rice. Treatments: uninoculated control (Ck), CdCl_2_, Bacteria, Bacteria + CdCl_2._

After 7 days of exposure to Cd stress, plants inoculated with bacterial strain showed enhanced growth as compared to uninoculated seedlings (Fig. 5). Cadmium stressed rice plants exhibited a substantial reduction in the shoot length and shoot fresh and dry weight as compared to the control plants. A reduction of 20%, 21%, and 77% were observed respectively (Table 3). The inoculation of bacterial strain, in the presence of Cd stress, showed a rise of 11% in the shoot length and a decline of 14% in the shoot fresh weight while 31% reduction was detected in the shoot dry weight, while bacteria alone showed 15% increase in shoot length, 57% in fresh weight, and an 8% increase in dry weight relative to control. Cd + bacteria showed a 27% increase in root length. Stressed plants showed 28% decrease in the root length relative to control plant. Cd + bacteria showed 27% increase in root length. The fresh and dried weight of roots was considerably improved in the bacterial inoculated plants as compared to the non-inoculated control plants. The stressed plants showed 67% and 65% reduction in the fresh and dry weight of roots as compared to the control plant. Bacteria (alone) showed an increase of 67% in the root fresh and 50% increase in root dry weight as compared to the control plants. Combination (Cd + bacteria) showed 33% increase in fresh and dry root weight as compared to the control plants (Table 3).

**Table 3:** Effect of bacterial inoculation on shoot length, shoot fresh and dry weight, root length, root fresh and dry weight of rice under Cd stress.

Treatments	Shoot length (cm)	Shoot fresh weight (g)	Shoot dry weight (g)	Root length (cm)	Root fresh weight (g)	Root dry weight (g)
Control	31.00 ± 1^b^	0.14 ± 0.01^b^	0.13 ± 0.05^a^	10.5 ± 0.44^b^	0.03 ± 0.01^b^	0.02 ± 0.01^a^
CdCl_2_	24.8 ± 1.6^c^	0.11 ± 0.00^c^	0.03 ± 0.02^c^	7.50 ± 0.88^c^	0.01 ± 0.01^c^	0.007 ± 0.0^b^
Bacteria	35.6 ± 1.4^a^	0.22 ± 0.02^a^	0.14 ± 0.01^a^	12.3 ± 1.02^a^	0.05 ± 0.01^a^	0.03 ± 0.02^a^
CdCl_2_ + Bacteria	34.5 ± 1.0^a^	0.16 ± 0.01^b^	0.09 ± 0.01^b^	13.3 ± 1.53^a^	0.04 ± 0.01^ab^	0.03 ± 0.01^a^

**Note:**

Different letters indicate a significant difference (*P* < 0.05) among the treatments.

#### Root scanning

**Figure 6:**
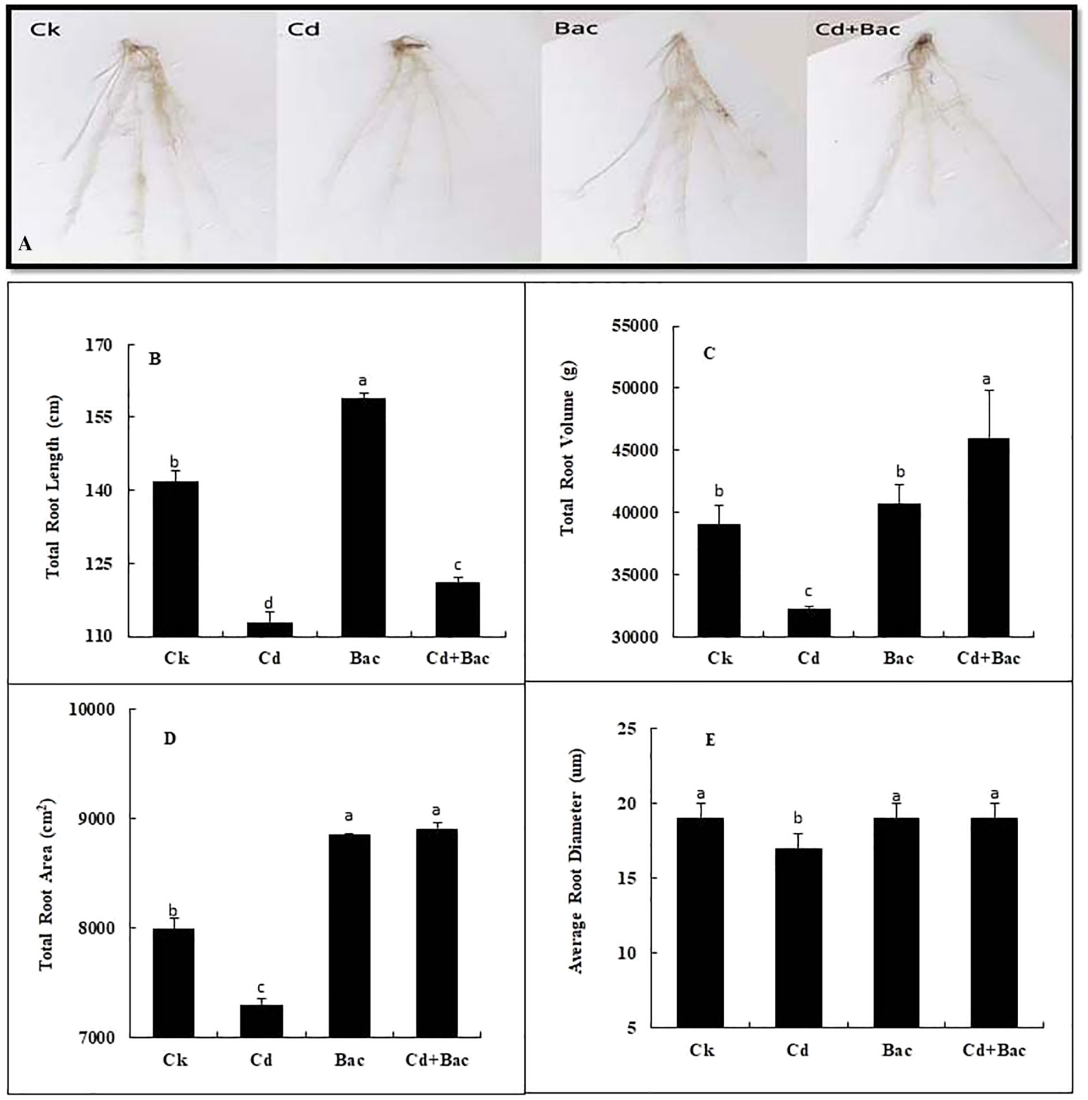
Effect of *B. cereus* S_6_D_1–105_ inoculation on root morphology. (A) root scanning images of different treatments, (B) total root length, (C) total root volume, (D) total root area, (E) average root diameter. Values are means of three replicates (*n* = 3).

Plants grown with bacterial strain exhibited a significant increase in the total root volume, root length, root area, and average diameter as compared to the Cd-treated plants (Fig. 6A). An 11% and 14% decrease was observed in total root length when plants were exposed to bacteria alone and Cd + bacteria treatment as compared to the control plants. Moreover, 20% decrease in total root length was observed in Cd stressed plants as compared to the control (Fig. 6B). A pronounced reduction of 17% in total root volume was noticed under Cd stress as compared to the control, while 4% and 17% increase in total root volume was observed in bacteria alone and Cd + bacteria treatment as compared to the control (Fig. 6C). Compared to control, the total root area of Cd-treated plants was decreased by 9% while 11% and 12% increase was noticed under alone bacteria and Cd + bacteria treatment (Fig. 6D). About 1% and 2% increase were observed for average diameter as compared to the control. Cadmium stressed plants showed 12% decrease in average root diameter as compared to the control plants (Fig. 6E).

#### Effect of bacterial inoculation and Cd stress on chlorophyll contents, malondialdehyde and relative water content

**Figure 7:**
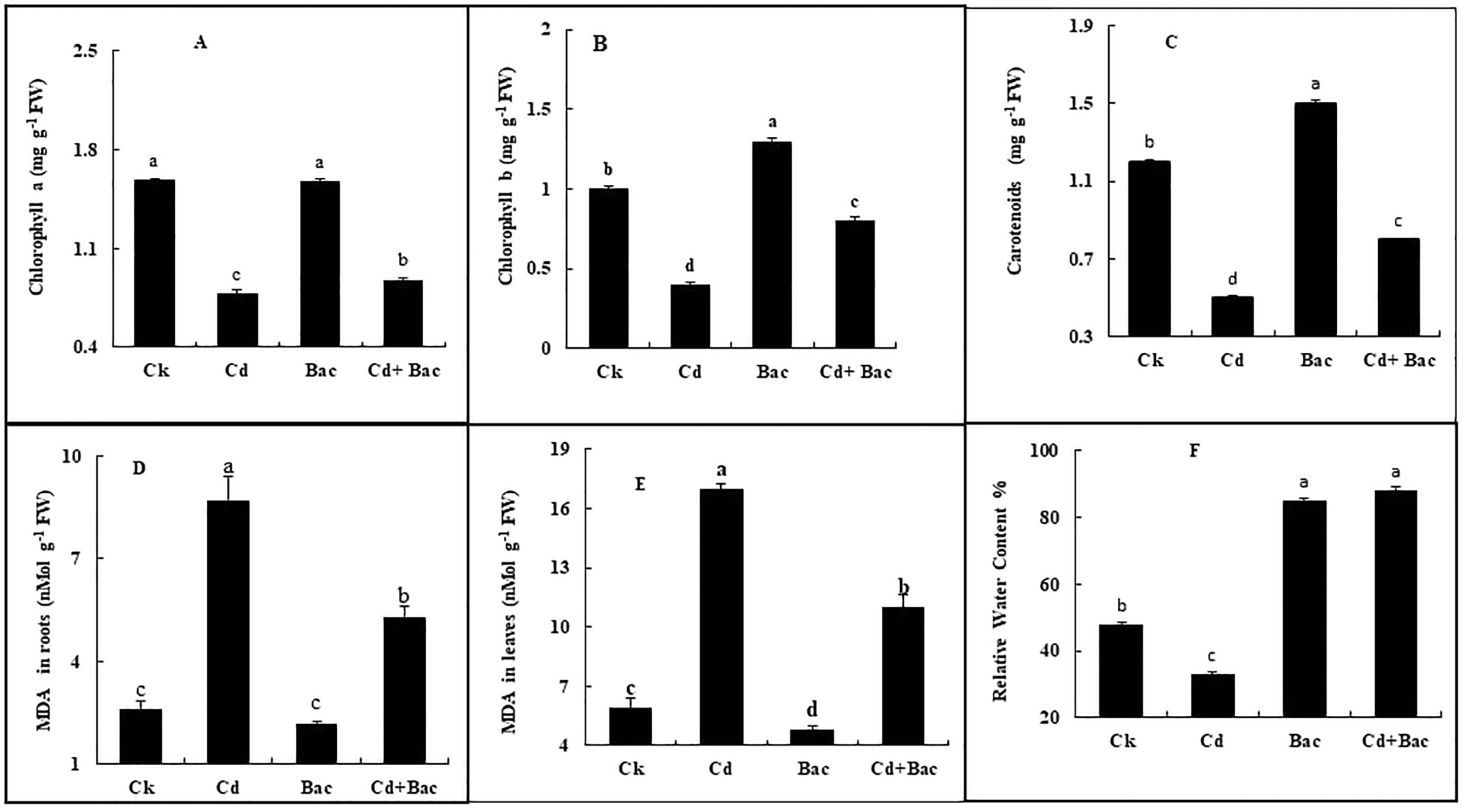
Effect of *B. cereus* S_6_D_1–105_ inoculation and Cd on (A) chlorophyll a, (B) chlorophyll b, (C) carotenoid, (D) malonaldehyde (MDA) content in the roots, (E) malonaldehyde (MDA) content in leaves, (F) relative water content of rice plants. Values are means of three replicates (*n* = 3).

Chlorophyll a, b, and carotenoid content was increased in bacteria treated plants by 6%, 39%, and 26% respectively, while Cd treated plants showed a decrease of 50%, 58%, and 44%, respectively. In Cd + bacteria inoculated plants, a decrease of 44%, 32%, and 43% was observed as compared to the control plants (Figs. 7A–7C). An increase of 229% in roots and 187% in leaves was observed in Cd treated plants while MDA levels in bacteria inoculated plants showed a reduction of 53% in roots and 18% in leaves. Whereas Cd + bacteria treated plants showed a 100% increase in MDA level in roots and 84% in leaves in comparison to the control plants (Figs. 7D and 7E). Under Cd stress, relative water content (RWC) of leaves was decreased. Inoculated plants with bacteria showed an increase in RWC in comparison to the control plant. Plants treated with bacteria (alone) displayed a 78% increase in RWC as compared to the control plants (Fig. 7F). Cadmium + bacteria treated plants had a higher (83%) leaves water content as compared to the control plants. Plants grown under Cd stress showed a 31% decrease in RWC as compared to the control.

#### Effect of bacterial inoculation and Cd stress on antioxidant enzymes

**Figure 8:**
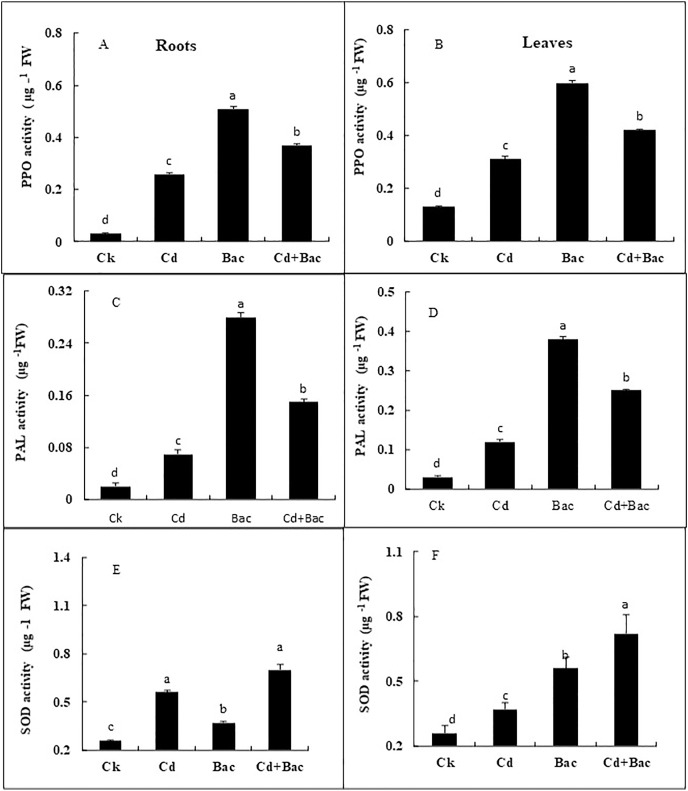
Effect of *B. cereus* S_6_D_1–105_ inoculation and Cd stress on antioxidant enzymes (PPO, PAL, and SOD) contents in roots (A) and leaves (B) of Super Basmati rice plants exposed to Cd stress.

A significant impact of bacterial strain S_6_D_1–105_ was observed on the PPO, PAL, and SOD activity (Figs. 8A–8F). Under Cd stressed conditions, PPO content increased by 766% in roots and 361% in leaves as compared to control plants, while PPO content in bacteria inoculated plants increased by 1,566% in roots and 361% in leaves as compared to the control, in Cd + bacteria inoculated plants an increase of 1,133% in roots and 223% in leaves was observed as compared to control plants (Figs. 8A and 8B). The PAL activity under Cd stress showed a 200% and 300% increase in roots and leaves respectively. In bacteria inoculated plants, an increase of 11,150% in both roots and leaves, was observed, while Cd + bacteria treatment caused a significant increase of 700% in roots and 657% in leaves in comparison to the control plants (Figs. 8C and 8D). In Cd stressed plants, SOD activity was increased by 215% in the leaves and 142% in the roots relative to the control plants. The SOD activity was considerably greater in bacteria inoculated plants in comparison to the control plants. Under Cd + bacteria treatment, SOD level was increased by 230% in leaves and 276% in roots, while under alone bacteria treatment its level was increased by 144% in leaves and 215% in roots (Figs. 8E and 8F).

#### Effect of bacterial inoculation and Cd stress on antioxidant enzymes (CAT, POD and APX) in rice plants

**Figure 9:**
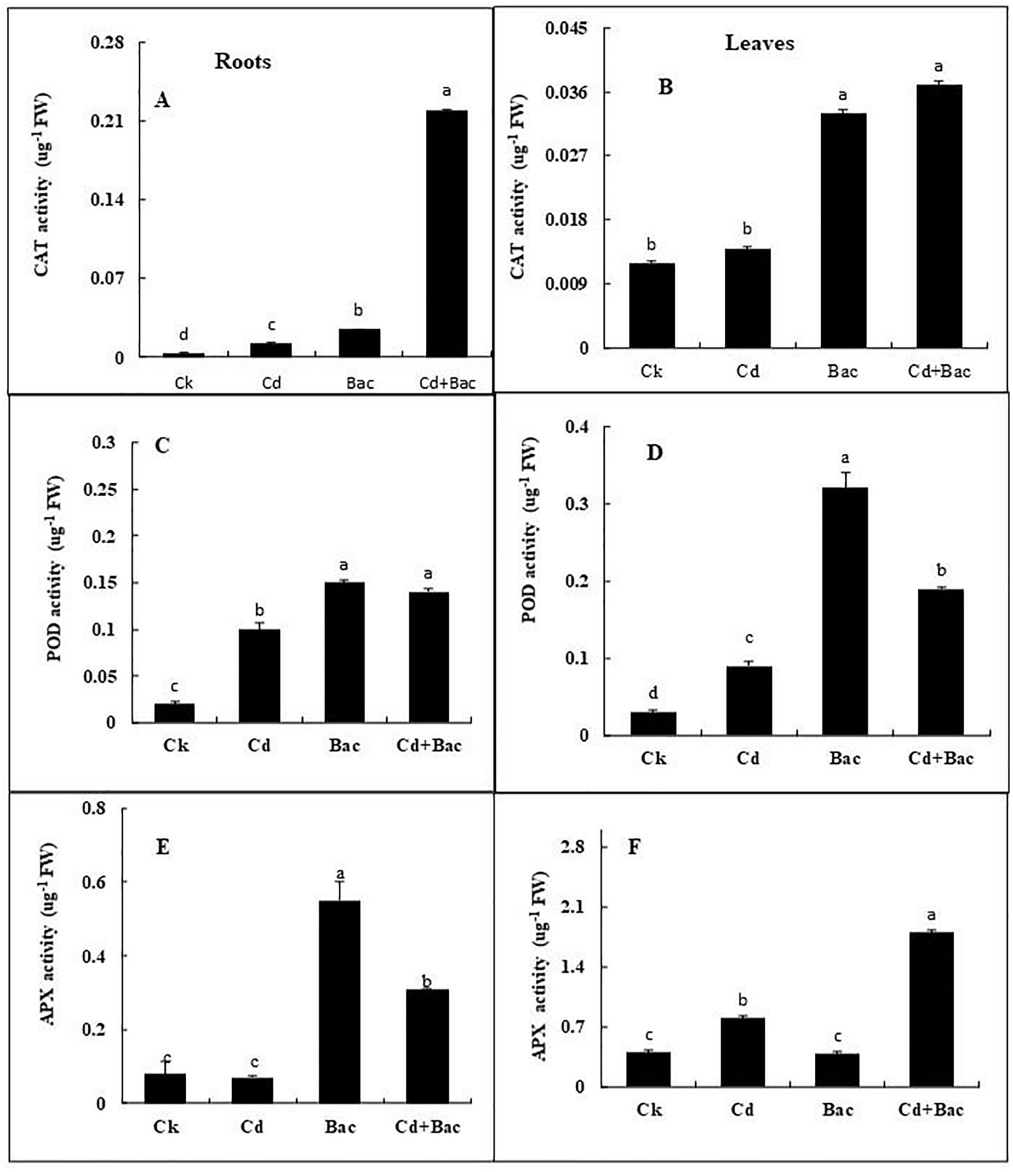
Effect of *B. cereus* S_6_D_1–105_ inoculation and Cd stress on antioxidant enzymes (CAT, POD, and APX) contents in roots (A) and leaves (B) of Super Basmati rice plants exposed to Cd stress.

**Figure 10:**
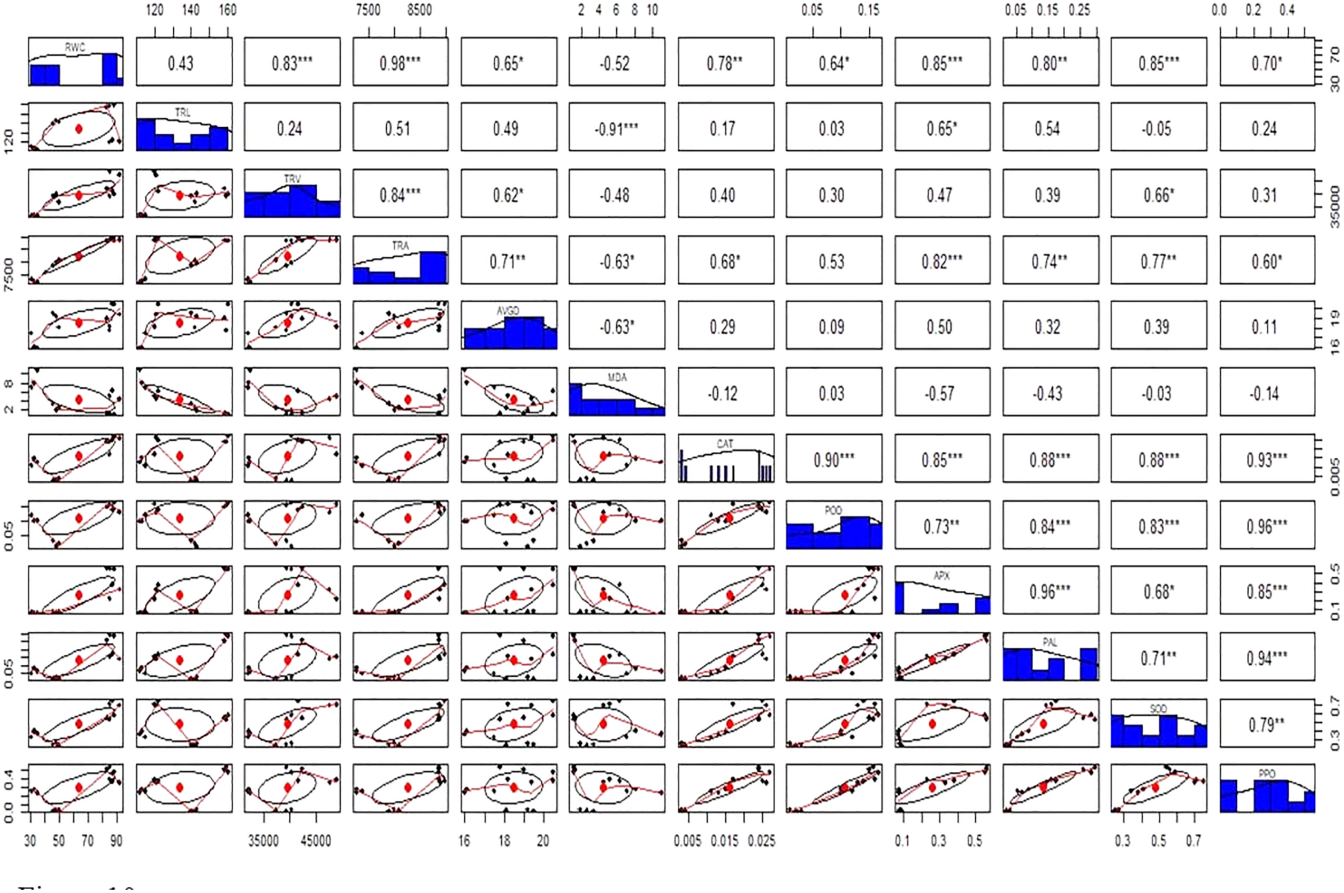
Relationship among roots relative water content (RWC), total root length (TRL), total root volume (TRV), total root area (TRA), average root diameter (AVGRD), malonaldehyde (MDA), catalase (CAT), peroxidase (POD), ascorbate peroxidase (APX), phenylalanine ammonia lyase (PAL), superoxide dismutase (SOD), and polyphenol oxidase (PPO) of Super Basmati rice plants under different treatments. Above the diagonal line showed correlation coefficient and below the diagonal line showed the bivariate. *p < 0.05, **p < 0.01, ***p < 0.001.

**Figure 11:**
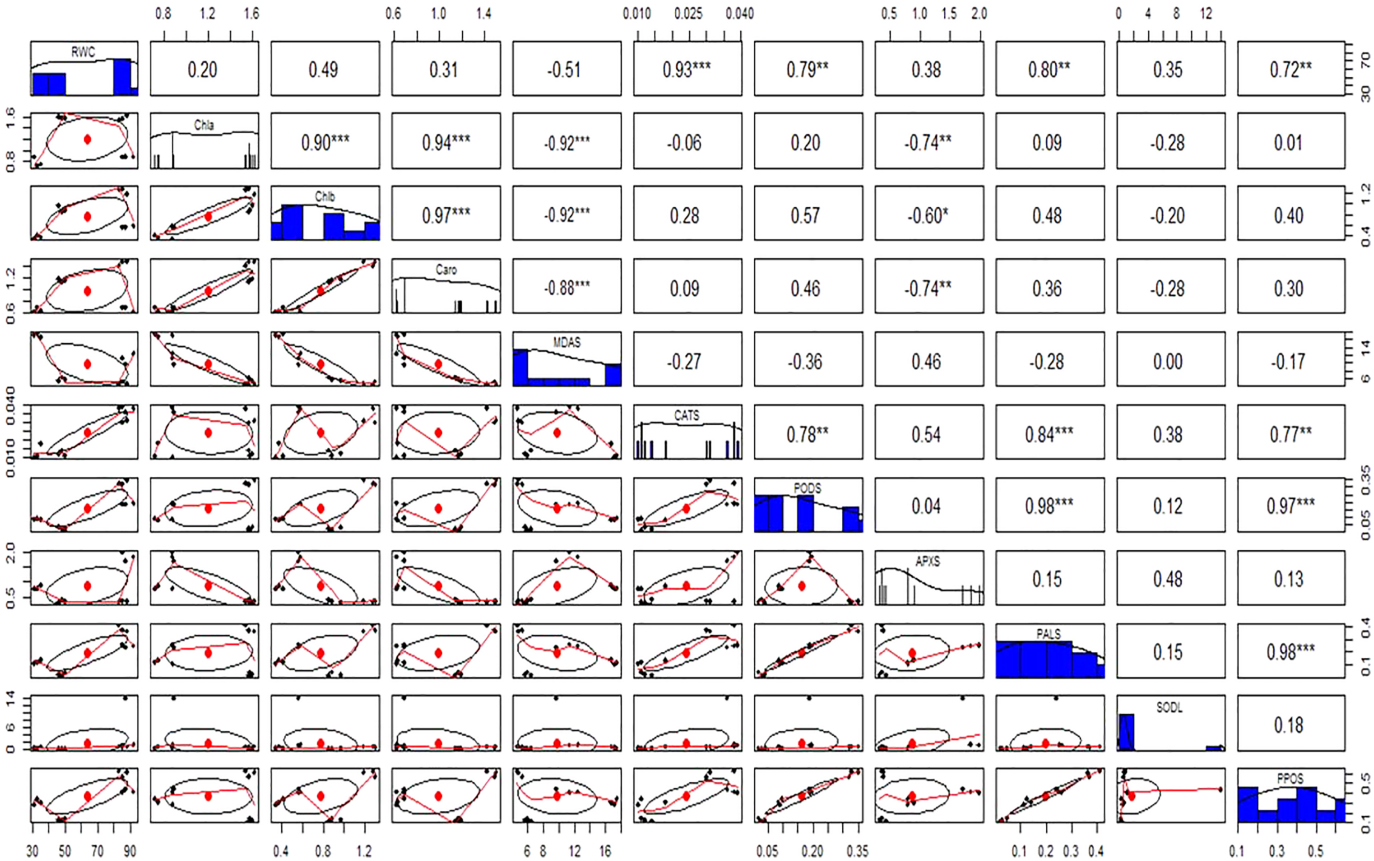
Relationship among leaves relative water content (RWC), chlorophyll (Chla), chlorophyll b (Chlb), carotenoid (Caro), malonaldehyde (MDA), catalase (CAT), peroxidase (POD), ascorbate peroxidase (APX), phenylalanine ammonia lyase (PAL), superoxide dismutase (SOD), and polyphenol oxidase (PPO) under different treatments. Above the diagonal line showed correlation coefficient and below the diagonal line showed the bivariate. *p < 0.05, **p < 0.01, ***p < 0.001.

Antioxidant enzymes (CAT, POD, and APX) activities were increased in stress-treated plants compared to the control (Figs. 9A–9F). CAT activity showed a 251% increase in Cd stressed plants in roots and a 15% increase in leaves. In bacterial inoculated plants, the CAT level was increased by 575% in roots and 175% in leaves. Under the Cd + bacteria treatment, CAT activity was increased by 5,981% in roots and 208% in leaves (Figs. 9A and 9B). POD level was increased by 400% in roots and 200% in leaves under Cd stress. In bacteria inoculated plants, POD level was increased by 650% in roots and 1,000% in the leaves in comparison to the control plant, while in Cd + bacteria inoculated plants, POD level was increased by 600% in roots and 533% in leaves compared to the control plants (Figs. 9C and 9D). APX activity was decreased in Cd treated plants by 84% in roots while an increase of 100% was recorded in leaves. Bacteria inoculated plants showed a 638% rise in roots APX and a 5% decrease in leaves APX, while Cd + bacteria inoculated plants showed a 359% increase in roots and a 350% increase in leaves APX as compared to their control plants (Figs. 9E and 9F). Physiological parameters of roots showed a strong positive correlation with antioxidant enzymatic activities, while MDA content showed a negative correlation with physiological and enzymatic parameters as depicted in Fig. 10. Leaves RWC showed a strong positive correlation with CAT, PAL, and PPO. Chlorophyll a content showed a strong positive correlation with chlorophyll b and carotenoids while a negative correlation was observed with MDA, CAT APX, and SOD. Chlorophyll b showed a positive correlation with carotenoid while chlorophyll b and carotenoid showed a negative correlation with MDA, APX, and SOD. CAT showed a positive correlation with POD, PAL, and PPO. POD showed a positive correlation with PAL and PPO, whereas PAL showed a positive correlation with PPO (Fig. 11).

## Discussion

Heavy metal contamination, resulting from naturally occurring processes or anthropogenic activities, has polluted the environment, and has become a serious cause of concern among majority of the countries around the world. Cadmium is ranked as 7th among the top 20 toxic metals (Sun et al., 2019) and is a severe threat to our environment due to its rising accumulation in agricultural soils through various components of irrigation, both indoor and outdoor (Anjum et al., 2016). In plants, Cd phytotoxicity results from Cd-induced oxidative stress. Oxidative stress impairs lipids, nucleic acids, and proteins, which in turn inhibits growth in the plant and can even cause cell death (Loix et al., 2017). In living organisms, cadmium occupies higher toxicity and mobility as compared to other harmful heavy metals (Li et al., 2017).

Numerous procedures, such as ion exchange, adsorption, or coagulation-flocculation, are available to remove Cd from Cd-contaminated regions. However, they are expensive and require high amounts of energy consumption as they rely on transforming the soil into different phases (Jan et al., 2019). Bioremediation, which involves the use of microorganisms to break down or removes environmental pollutants from a given area, is a much more economical and eco-friendly method of reducing Cd contamination. Plants, plant-associated bacteria, and heavy metal resistant bacteria inhabiting extreme environments (such as Cd-contaminated soils) are likely to be more tolerant to the high level of Cd stress (Ahmad et al., 2014). Furthermore, many of the Cd resistant PGP that can be cultured have been discovered from regions susceptible to Cd, such as the waste products of industries that contain Cd (Pandey et al., 2010). In a previous study, it was noted that bacteria isolated from the environments that has been polluted by a heavy metal show greater tolerance towards the heavy metals stress (Pál et al., 2005; Mitra et al., 2018). In addition to this, heavy metal resistant PGPB also provides a suitable replacement or reduction in the use of chemical fertilizers, pesticides, and nutritional supplements in agriculture (Jan et al., 2019).

This study aimed to identify the sources that have toxicity-ameliorating attributes and consequently improve the rice variety grown under heavy metal stress. Eleven industrial strains of bacteria were isolated and screened for tolerance against Cd. The novelty of the screening was attributed to different concentrations of Cd (100 µM and 200 µM). Only three bacterial strains grew under Cd stress. These three isolates were further screened by determining the MIC of Cd after which one isolate, S_105_, was selected for further experimentation as it had the highest MIC value (1,000 µM). The assessment is important not just because of the need to measure resistance trend, but more importantly, the MIC value is correlated to the dosage required for effective therapy (Huang et al., 2015).

Furthermore, the morphological attributes and PGP traits of the selected bacterial strain were determined. S_6_D_1–105_ strain *of B. cereus* was able to solubilize potassium and phosphate and synthesize protease and siderophore. Höfte et al. (1994) noted that in *Pseudomonas aeruginosa* the enzyme recombinase regulates the zinc-induced production of siderophore. Zinc and Cd are both divalent cations thus it is predicted that Cd-induced siderophore production is controlled by an analogous mediator (Sinha & Mukherjee, 2008). Moreover, Naveed et al. (2014) reported that solubilizing phosphate improves shoot and root biomass thereby increasing yield amount under both stressed and normal conditions (Mitra et al., 2018). Under stress conditions, phosphate uptake by plants is reduced (Mitra et al., 2018) however, PGPB that can solubilize phosphate greatly increase its uptake by plants (Zaidi et al., 2006).

Cadmium uptake and tolerance by plants vary greatly between inter-species and intra-species. In a previous study, He et al. (2008), reported that rice seeds of different genotypes under Cd treatment exhibited significantly varying Cd sensitivity. In the present study, three rice genotypes, Basmati 515 super basmati, and Chenab were exposed to various concentrations of CdCl_2_. Super basmati was selected for further experimentation as it showed the greatest tolerance against Cd contamination. In Li et al. (2003) study, it was stated that the rice plant’s ability to uptake Cd is reliant on its root activities and the rhizosphere environment (He et al., 2008).

Rice inoculated with bacteria showed improved growth and biomass under Cd stress. This improvement was determined by comparing the length, fresh weight, and dry weight of the rice leaves and roots under different conditions. Rice plants that were not inoculated with the bacteria showed a decrease in these parameters when exposed to Cd stress, as compared to the control. Previously, it was reported that bacterial inoculation by S8 strain could enhance the agronomic traits under Cd stress (Mitra et al., 2018). Monocotyledonous plants, like rice, have a predominant fibrous root system that enables the plant to anchor and gather water efficiently. Improved shoot elongation and fibrous roots signified that bacterial strain S_105_ was effectively able to elevate rice growth in response to Cd contamination. In the present experiment, the RWC was higher in rice inoculated with bacteria and lowest in Cd-treated plants as compared to the control. Measuring RWC indicates the stress response of plants. It was reported that the RWC of PGPB-treated plants was higher than that of the control during abiotic stress (Singh et al., 2013). Thus, showing the beneficial association can undermine such stresses and enhance plant growth. The PGPR-inoculated plants not only reduce stress but also help to fetch higher water quantity from sources inaccessible to control plants (Kang et al., 2014). The chlorophyll content is directly associated with the rate of photosynthesis thus making it an essential growth parameter (Mitra et al., 2018). Photosynthetic pigments, for example, chlorophyll-a or chlorophyll-b, are fundamental for photosynthesis as they play a crucial role in the photosystem II light-harvesting complex thus making them vital for the plant’s chances of survival. The most commonly observed symptom of Cd phytotoxicity has been the loss of chlorophyll (chlorosis) (Chao, Hong & Kao, 2010) as Cd is a known inhibitor of chlorophyll synthesis. Cadmium reduces the efficiency of the carbon assimilation pathway in the chloroplast and disrupts the Chlorophyll content and protein complexes (Jan et al., 2019). In the present study, Chlorophyll contents were affected by different concentrations of Cd. However, inoculation of plants with the selected bacterial strain substantially increased chlorophyll content in both normal and Cd-treated plants. Similar results have been observed in previous studies (Jan et al., 2019). Cadmium stress caused a significant increase in the levels of MDA produced by the plants. Malondialdehyde, is known as an extended parameter of oxidative stress, and is the final product of lipid peroxidation, and severely damages plant cell membranes through peroxidation of the lipids present in the membrane subsequently disrupting the processes of respiration and photosynthesis in plants. Malondialdehyde content can be used to evaluate the extent of peroxidation of the membrane lipids and their environmental stress response (Chen, 1991). Bacterial inoculation notably reduced MDA contents in the rice plants as compared to uninoculated plants. Application of bacterial strains K5 (Pal & Sengupta, 2016) and S8 (Mitra et al., 2018) in rice seedlings subjected to Cd stress supported the current findings. Lowered levels of MDA content reduce cellular membrane damage, thus conserving cellular integrity (Pramanik et al., 2018). Another toxic effect induced by heavy metals stress in plants is the imbalance of redox homeostasis which results in progressive oxidative damage that can lead to cell senescence and even cell death (Lin et al., 2012). Reactive oxygen species (ROS), such as hydrogen peroxide or superoxide, are responsible for this oxidative damage (Apel & Hirt, 2004). Although Cd is a non-redox active metal ion and thus unable to generate ROS, several reports of oxidative damage being induced in organisms exposed to Cd have been documented (Jabeen et al., 2021; Cuypers et al., 2010). To counter the Cd-induced overproduction of ROS and thereby minimize oxidative stress, plants generate antioxidative ROS scavenging systems. These scavengers can be enzymatic (SOD, CAT, PPO, PAL, POD, and APX) or non-enzymatic (ascorbate, proline, and glutathione). SOD is a metalloenzyme that acts as the first line of defense against plant stress (Hussain et al., 2015). SOD catalyzes the detoxification of superoxide anions into hydrogen peroxide (H_2_O_2_) which is further broken down into water and oxygen by CAT and glutathione peroxidases (Cuypers et al., 2010). However, persistent exposure to Cd and other abiotic stresses can transmute the efficient endogenous mechanisms plants use for reducing oxidative damage (Pramanik et al., 2018). Several prior studies have reported multiple oxidative stress-reducing PGPR strains that have reduced oxidative damage in plants (Pandey & Bhatt, 2016). In the present study, all the antioxidant enzymes showed a significant increase in activity in Cd resistant PGPB inoculated plants as compared to Cd stress thus suggesting that PGPB inoculation reduced the oxidative stress of the rice plants.

## Conclusions

Hydroponically grown rice plants, inoculated with *B. cereus* strain S_6_D_1–105_ were able to show improved plant growth, increased plant biomass, chlorophyll contents, relative water content, different antioxidant enzymatic activity such as catalase, superoxide dismutase, ascorbate peroxidase, peroxidase, polyphenol oxidase, and phenylalanine ammonia-lyase, and reduced MDA content in both roots and leaves of rice plant under Cd stress. Our results showed that the *B. cereus* S_6_D_1–105_ can be used as a biofertilizer which might be beneficial for rice cultivation in Cd polluted soils.

## Supplemental Information

10.7717/peerj.13131/supp-1 Supplemental Information 1 Raw data.

## Additional Information and Declarations

### DNA Deposition

The following information was supplied regarding the deposition of DNA sequences: The partial sequence for *Bacillus cereus* strain is available at GenBank: OK310861.1.

### Data Availability

The following information was supplied regarding data availability: The raw data are available in the Supplemental File.
